# Investigating the in vitro antibacterial efficacy of composite bone cement incorporating natural product-based monomers and gentamicin

**DOI:** 10.1186/s13018-024-04646-7

**Published:** 2024-03-06

**Authors:** Yu-Chen Kan, Rui Guo, Yang Xu, Lu-Yang Han, Wen-Han Bu, Long-Xu Han, Jian-Jun Chu

**Affiliations:** 1 Department of Orthopedics, The Second People’s Hospital of Hefei, Hefei Hospital, Affiliated to Anhui Medical University, No. 246 of Heping Road, Yaohai District, Hefei, Anhui 230011 China; 2 The Fifth Clinical Medical School of Anhui Medical University, Hefei, Anhui 230032 China; 3 School of Food and Biological Engineering, Hefei University of Technology, Hefei, Anhui 230009 China

**Keywords:** Antibacterial, Biocompatibility, Bone cement, Infection, Natural product extracts

## Abstract

**Objective:**

The objective of this study is to investigate the impact of four natural product extracts, namely, aloe-emodin, quercetin, curcumin, and tannic acid, on the in vitro bacteriostatic properties and biocompatibility of gentamicin-loaded bone cement and to establish an experimental groundwork supporting the clinical utility of antibiotic-loaded bone cements (ALBC).

**Methods:**

Based on the components, the bone cement samples were categorized as follows: the gentamicin combined with aloe-emodin group, the gentamicin combined with quercetin group, the gentamicin combined with curcumin group, the gentamicin combined with tannic acid group, the gentamicin group, the aloe-emodin group, the quercetin group, the curcumin group, and the tannic acid group. Using the disk diffusion test, we investigated the antibacterial properties of the bone cement material against *Staphylococcus aureus* (*n* = 4). We tested cell toxicity and proliferation using the cell counting kit-8 (CCK-8) and examined the biocompatibility of bone cement materials.

**Results:**

The combination of gentamicin with the four natural product extracts resulted in significantly larger diameters of inhibition zones compared to gentamicin alone, and the difference was statistically significant (*P* < 0.05). Except for the groups containing tannic acid, cells in all other groups showed good proliferation across varying time intervals without displaying significant cytotoxicity (*P* < 0.05).

**Conclusion:**

In this study, aloe-emodin, quercetin, curcumin, and tannic acid were capable of enhancing the in vitro antibacterial performance of gentamicin-loaded bone cement against *S. aureus*. While the groups containing tannic acid displayed moderate cytotoxicity in in vitro cell culture, all other groups showed no discernible cytotoxic effects.

## Introduction

Postoperative prosthetic infection is one of the most challenging complications in orthopedic surgery. [1, 2] A crucial aspect of the management of postoperative prosthetic infections is the utilization of antibiotic-loaded bone cement (ALBC) as a void filler subsequent to surgical debridement to control infection. Gentamicin is the most commonly loaded antibiotic in ALBC [3]. It is extensively used in ALBC due to its high thermal stability—a relatively uncommon feature among available antibiotics—coupled with its broad-spectrum antibacterial properties and low cost [4]. However, in recent years, researchers have found that gentamicin adversely affects osteoblast activity [5]. Therefore, the issue of reducing the concentration of gentamicin in bone cement deserves attention. [6, 7].

It has been demonstrated in recent pharmacological studies that combining natural product extracts with antibiotics can significantly enhance the antibacterial effects of antibiotics. [8, 9] Deepika et al. [10] observed heightened antibiofilm activity against multidrug-resistant *Pseudomonas aeruginosa* (MDR-PA) with a combination of gentamicin and the flavonoid rutin when compared to gentamicin used alone. Zhou et al. reported that the combination of gentamicin and andrographolide could significantly inhibit the biofilm formation of *Staphylococcus aureus* [11]. 

In this study, we selected four natural product extracts with prior documented potential to augment the antibacterial effect of gentamicin and incorporated these into the bone cement that we used [12]. We investigated the effects of these four natural product extracts on the antibacterial activity and cytotoxicity of gentamicin-loaded bone cements.

## Materials and methods

### Materials and reagents

All PMMA bone cement samples were composed of polymethyl methacrylate (PMMA) bone cement powder and liquid methyl methacrylate (MMA) (purchased from SYNIMED s.a.r.l. (France) Eurofix bone cement) monomer in a ratio of 40 g: 20 mL. We procured *S. aureus* (ATCC 25,923) from The Second People’s Hospital of Hefei, China. This study was approved by the Biomedical Ethics Committee of Hefei University of Technology (approval no. HFUT20220522-001; approval date. “22 May 2022”).

For biocompatibility assessments, we utilized a solid-liquid extraction solution prepared using cylindrical bone cement samples measuring 6 mm in diameter and 12 mm in height. We conducted bacteriostatic experiments on the bone cement using disc-shaped samples measuring 6 mm in diameter and 3 mm in height.

### Grouping for experiments

#### Test group

The mixtures were constituted based on different ratios of PMMA bone cement powder to natural product extract to gentamicin, as follows: 40 g: 2 g (5 wt%): 0 g (0 wt%); 40 g: 2 g (5 wt%): 1 g (2.5 wt%); 40 g: 2 g (5 wt%): 1.4 g (3.5 wt%); 40 g: 2 g (5 wt%): 1.8 g (4.5 wt%); and 40 g: 2 g (5 wt%): 2 g (5 wt%). We fabricated polytetrafluoroethylene disc-shaped molds and used these to prepare four groups of disc-shaped standard samples. The samples were divided into four categories based on the content of natural product extracts: the aloe-emodin groups (groups I, II, III, IV, and V), the quercetin groups (groups VI, VII, VIII, IX, and X), the curcumin groups (groups XI, XII, XIII, XIV, and XV), and the tannic acid groups (groups XVI, XVII, XVIII, XIX, and XX), with three specimens in each group (Table 1). These four natural product extracts were all produced by Macklin Company, and purchased from Guangzhou Qiyun Biotechnology Co., LTD.

**Table 1:** Composition ratios of bone cements loaded with antibiotics and TCM extracts (*n* = 3)

Group	Group number	Antibiotic content per 40 g of bone cement (g)	Antibiotic content (wt%)	TCM extract content per 40 g of bone cement (g)	TCM extract content (wt%)
Aloe-emodin group	I	0	0	2	5
	II	1	2.5	2	5
	III	1.4	3.5	2	5
	IV	1.8	4.5	2	5
	V	2	5	2	5
Quercetin group	VI	0	0	2	5
	VII	1	2.5	2	5
	VIII	1.4	3.5	2	5
	IX	1.8	4.5	2	5
	X	2	5	2	5
Curcumin group	XI	0	0	2	5
	XII	1	2.5	2	5
	XIII	1.4	3.5	2	5
	XIV	1.8	4.5	2	5
	XV	2	5	2	5
Tannic acid group	XVI	0	0	2	5
	XVII	1	2.5	2	5
	XVIII	1.4	3.5	2	5
	XIX	1.8	4.5	2	5
	XX	2	5	2	5
Sole gentamicin group (Control group)	XXI	1	2.5	0	0
	XXII	1.4	3.5	0	0
	XXIII	1.8	4.5	0	0
	XXIV	2	5	0	0

#### Control group

For the mixtures of the control groups (groups XXI, XXII, XXIII, and XXIV), the ratios of PMMA bone cement powder to gentamycin sulfate (produced by Macklin Company, purchased from Guangzhou Rentai Technology Co., LTD) were 40 g: 1 g (2.5 wt%), 40 g: 1.4 g (3.5 wt%), 40 g: 1.8 g (4.5 wt%), and 40 g: 2 g (5 wt%). The control groups comprised 12 specimens (Table 1).

### Preparation of the drug-loaded bone cement

We evenly mixed the PMMA bone cement power, corresponding natural product extracts (aloe-emodin, quercetin, curcumin, and tannic acid), and gentamycin sulfate as per the mentioned ratios under sterile and light-devoid conditions at room temperature (23℃ ± 1℃). The mixture in the control group did not contain natural product extracts. We then mixed the bone cement powder with the liquid phase of bone cement using a ratio of 40 g of powder to 20 mL of liquid. The resulting mixture was then thoroughly stirred until a uniform consistency was achieved. In the “dough phase,” the resultant mixture was placed into a disc-shaped mold made of polytetrafluoroethylene, which was developed by our team. We applied appropriate pressure to thoroughly compress the bone cement mixture to avoid forming discontinuous layers. Thirty minutes later, we extracted the samples from the mold with the help of a demolding assistive rod. The samples were all standard disc-shaped bone cement specimens, each measuring 6 mm in diameter and 3 mm in height.

### Antibacterial experiment (zone of inhibition test)

We examined the antibacterial activity of bone cement against *S. aureus*. We uniformly spread 100 µL of a *S. aureus* suspension with a McFarland turbidity of 0.5 on agar plates. The bone cement specimens used in the experiment, prepared using polytetrafluoroethylene molds, measured 6 mm in diameter and 3 mm in height. The specimens were placed on each agar plate such that the center-to-center distance between each sample was greater than 24 mm and the distance between the sample and the edge of the agar plate was more than 15 mm. The specimens were then incubated on the agar plates at 37 °C for 24 h. We evaluated the antibacterial activity by measuring the zones of inhibition formed around the bone cement specimens.

### Cytotoxicity (assessed with the CCK-8 test using osteoblast precursor cells [MC3T3E1] of mouse embryos)

#### Grouping for the experiments

We mixed PMMA bone cement powder, natural product extracts, and gentamicin as per the specified ratios. We obtained four categories of standard circular bone cement specimens from molding the resulting mixture using polytetrafluoroethylene disc-shaped molds that we developed. We made the following groups based on natural product extracts: the aloe-emodin groups (groups I, II, and V), the quercetin groups (groups VI, VII, and X), the curcumin groups (groups XI, XII, and XV), the tannic acid groups (groups XVI, XVII, and XX), the group of sole gentamicin (control group XXIV), and the control group (Group XXV). Each group comprised three specimens (Table 2).

**Table 2:** Composition ratios of bone cements loaded with antibiotics and TCM extracts for cytotoxicity experiments

Group	Group number	Antibiotic content (wt%)	TCM extract content (wt%)
Aloe-emodin group	I	0	5
	II	2.5	5
	V	5	5
Quercetin group	VI	0	5
	VII	2.5	5
	X	5	5
Curcumin group	XI	0	5
	XII	2.5	5
	XV	5	5
Tannic acid group	XVI	0	5
	XVII	2.5	5
	XX	5	5
Sole gentamicin group (Control group)	XXIV	5	0
Culture medium group	XXV	0	0

#### Experimental method for cytotoxicity assay (CCK-8 method)

The cells (MC3T3-E1, purchased from Hunan Fenghui Biotechnology Co., LTD.) were inoculated into a 96-well cell culture plate at a concentration of 5 × 10^3^ per well and cultured in minimum essential medium (MEM) supplemented with 10% fetal bovine serum. Subsequently, the original cell culture medium was discarded, and 100 µL of test material extracts at different dilutions were co-cultured with MC3T3-E1 cells in triplicate. Cells cultured solely with 10% serum (i.e., cells not exposed to the test material) were used as the cell control. We observed and recorded indicators of osteoblasts on days 1, 3, and 5 to evaluate the in vitro biocompatibility of bone cement, including morphology changes of cell culture medium, optical density (OD) values, and relative growth rate (RGR). The absorbance of cell culture medium at 450 nm for each well was determined using the enzyme-linked immunosorbent assay (ELISA). We used the formula for calculating RGR:

$$\text{R}\text{G}\text{R}=\frac{{\text{O}\text{D}}_{\text{T}}-{\text{O}\text{D}}_{\text{R}}}{{\text{O}\text{D}}_{\text{N}}-{\text{O}\text{D}}_{\text{R}}}\times 100\text{\%}$$

In the formula, OD_T_ represents the absorbance of the test groups, OD_N_ represents the absorbance of the negative control groups, and OD_R_ represents the absorbance of the cell-free culture medium.

In adherence to stringent aseptic protocols, the MC3T3-E1 cells were aseptically extracted and subsequently introduced into pre-warmed double-distilled water at 37 °C for dissolution. The resulting cell suspension was then relocated to a laminar flow clean bench for subsequent utilization. Following the thawing process, the cell suspension was introduced into RPMI 1640 medium, comprising 10% fetal bovine serum and 1% penicillin-streptomycin solution, within a sterilized centrifuge tube. Subsequently, centrifugation was conducted at 1000 rpm for a duration of three minutes at ambient temperature. Cells at the base of the sterilized centrifuge tube were resuspended in one mL of medium, and evenly dispersed through gentle pipetting. Subsequently, this resuspended cell population was translocated into a cell culture flask that had been pre-supplemented with a standardized volume of culture medium, and cultured in a stable-temperature incubator, maintained at 37 °C with a 5% CO2 atmosphere. Regular medium replacement was undertaken at scheduled intervals, and concurrent observations were systematically conducted. Cell passaging was executed when cell confluence approached approximately 80%. MC3T3-E1 cells in the logarithmic growth phase were dissociated into single-cell suspensions using trypsin. These dissociated cells were then seeded at a volume of 100 µL per well in a 96-well plate and cultured for 24 h in a stable-temperature incubator, maintained at 37 °C with a 5% CO2 atmosphere. On days 1, 3, and 5 of the cell culture, 1/10 volume of CCK8 reagent was introduced to each well employing the CCK8 method. The respective wells were subsequently incubated in the incubator, maintained at 37 °C with a 5% CO2 atmosphere, for a duration of three hours. Subsequently, the cellular morphology within each experimental group was examined under a microscope. The OD of the cells was measured at a wavelength of 450 nm using an ELISA reader, and the RGR was calculated. Additionally, cellular morphological changes and cytotoxicity grading were assessed in accordance with the GB/T 16,886 standard [13].

### Statistical analysis

SPSS 25.0 software was used to analyze data pertaining to the inhibition zone, cell absorbance, and RGR. Quantitative data are represented as mean ± standard deviation ($\stackrel{-}{x }$± s). Statistical analysis was conducted using one-way analysis of variance (ANOVA). Statistical significance was defined as a *P*-value less than 0.05 (*P* < 0.05).

## Results

### Bacteriostatic effect of gentamicin-loaded bone cement specimens against *S. Aureus* at various concentrations

**Fig. 1:**
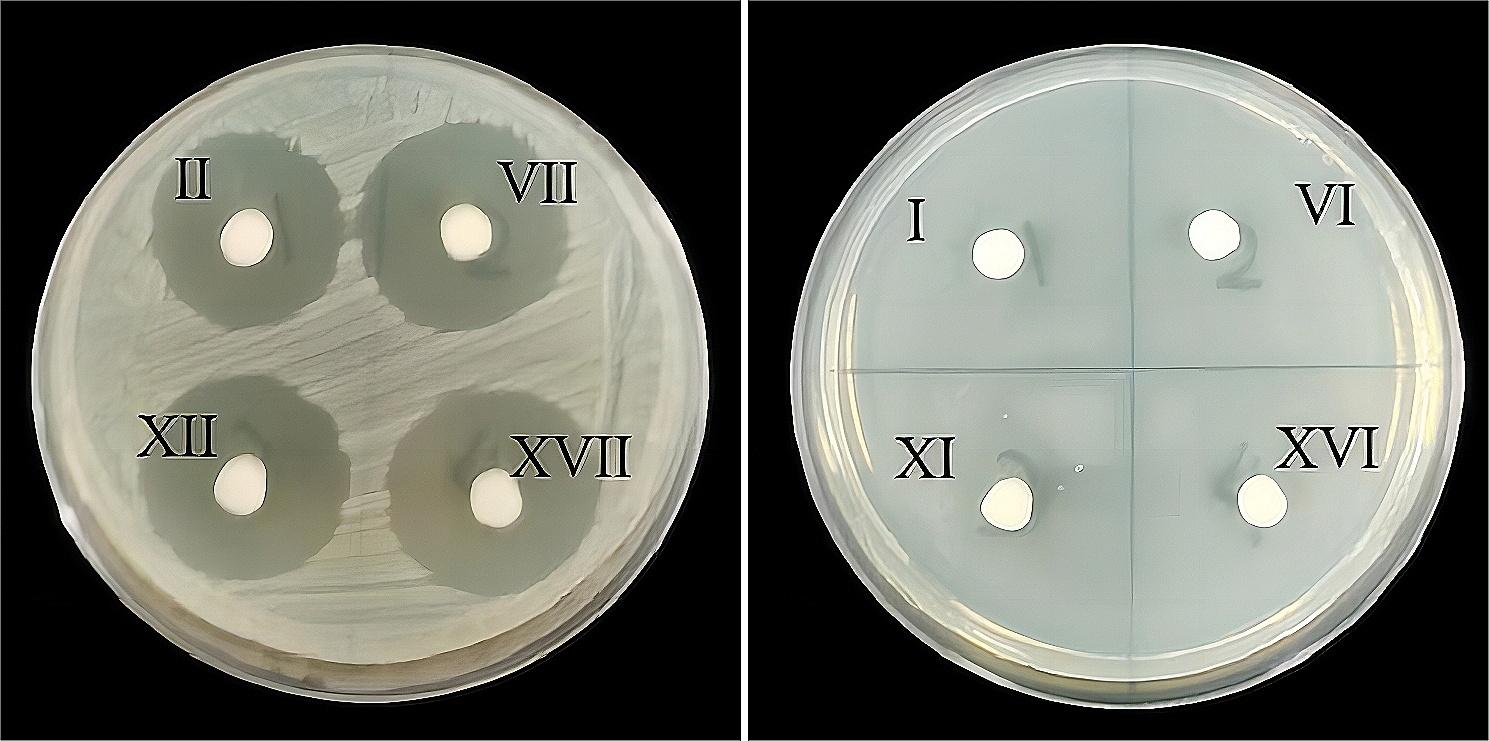
Diagram of experiments related to the zones of inhibition. *Note* Groups II, VII, XII, and XVII show inhibition zones against *Staphylococcus aureus* when the four natural product extracts combined with 2.5% gentamicin were used. Groups I, VI, XI, and XVI show inhibition zones against *Staphylococcus aureus* when the four natural product extracts were used in the absence of gentamicin. Natural product extracts alone do not demonstrate significant anti-*Staphylococcus aureus* properties

The experimental data and column charts pertaining to the experiment on the bacteriostatic effect of gentamicin-loaded bone cement specimens against *S. aureus* at varied concentrations yielded (with the natural product extract content consistently at 5%) are shown in Fig. 1. The results were as follows:

#### Intra-group comparison of natural product extracts

**Fig. 2:**
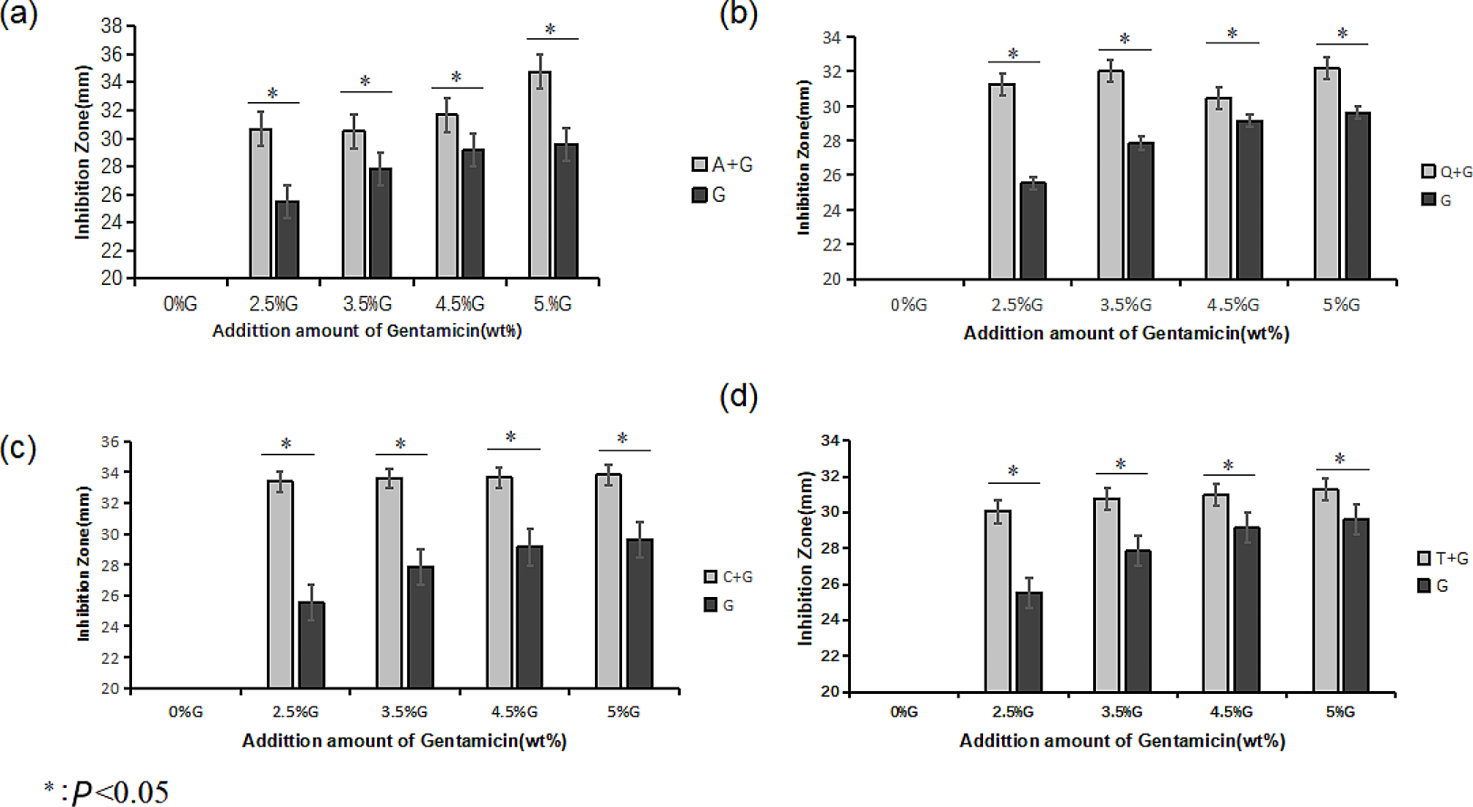
Experiments on inhibition zones of the four natural product extracts. **(a)** Aloe-emodin **(b)** Quercetin **(c)** Curcumin **(d)** Tannic acid. *Note* Denotations of each letter in Fig. 2: A: aloe-emodin, Q: quercetin, C: curcumin, T: tannic acid, G: gentamicin

We conducted a repeated measures ANOVA to compare the resultant inhibition zones between aloe-emodin and sole gentamicin at different concentrations. The main effect of concentration was significant (F = 1529.06, *P* < 0.05). An increase in concentration resulted in larger zones of inhibition. Similarly, the main effect for drug categories was also significant (F = 101.05, *P* < 0.05). As shown in Fig. 2, group II, the group of bone cement containing aloe-emodin and supplemented with 2.5% gentamicin, exhibited larger zones of inhibition when compared to group XXIV, the group of bone cement solely supplemented with 5% gentamicin. The interaction effect was also significant (F = 9.78, *P* < 0.05), indicating that different concentrations of aloe-emodin and sole gentamicin resulted in varied outcomes.

Similarly, we used repeated measures ANOVA for the comparison of inhibition zones between quercetin and sole gentamicin at different concentrations. The main effect of concentration was significant (F = 3118.85, *P* < 0.05). An increase in concentration resulted in larger zones of inhibition. The main effect for drug categories was also significant (F = 166.05, *P* < 0.05). As shown in Fig. 2, group VII, the group of bone cement with quercetin supplemented with 2.5% gentamicin, exhibited larger zones of inhibition compared to group XXIV, the group of bone cement solely supplemented with 5% gentamicin. Finally, the interaction effect was significant (F = 22.57, *P* < 0.05), indicating that quercetin and sole gentamicin resulted in varied outcomes at different concentrations.

In subsequent analyses, we obtained consistent findings pertaining to the size of inhibition zones for curcumin and tannic acid, similar to the previously observed results for aloe-emodin and quercetin. In particular, groups XII and XVII, the test groups supplemented with 2.5% natural product extracts, exhibited larger zones of inhibition compared to group XXIV, the group of bone cement solely supplemented with 5% gentamicin.

#### Comparison between the test groups (aloe-emodin, quercetin, curcumin, and tannic acid groups)

Using single-factor ANOVA, we compared the effects of different drugs on the zones of inhibition without considering concentration. The result was F = 0.24, *P* < 0.05. The differences in the size of the inhibition zones among different drugs did not reach statistical significance. Nevertheless, there were discernible differences among the groups, with the curcumin groups exhibiting the most extensive zones of inhibition, followed by the aloe-emodin, quercetin, and tannic acid groups in that order, while the gentamicin groups displayed the smallest zones of inhibition.

### Cell proliferation and toxicity experiments (in vitro experiments)

#### OD value, RGR, and toxicity grading

Cell OD Value: We measured the OD values of cells in each group on the 1st, 3rd, and 5th days of cell culture using the CCK-8 method. We compared the OD values of each group across varying time intervals pairwise using repeated measures ANOVA. We compared the groups of aloe-emodin combined with gentamicin (groups I, II, and V), the groups of quercetin combined with gentamicin (groups VI, VII, and X), the groups of curcumin combined with gentamicin (groups XI, XII, and XV), and the groups of tannic acid combined with gentamicin (groups XVI, XVII, and XX) with the sole gentamicin control group (group XXIV). We found statistically significant differences in OD values and cell RGR on the 1st, 3rd, and 5th days (*P* < 0.05).

**Fig. 3:**
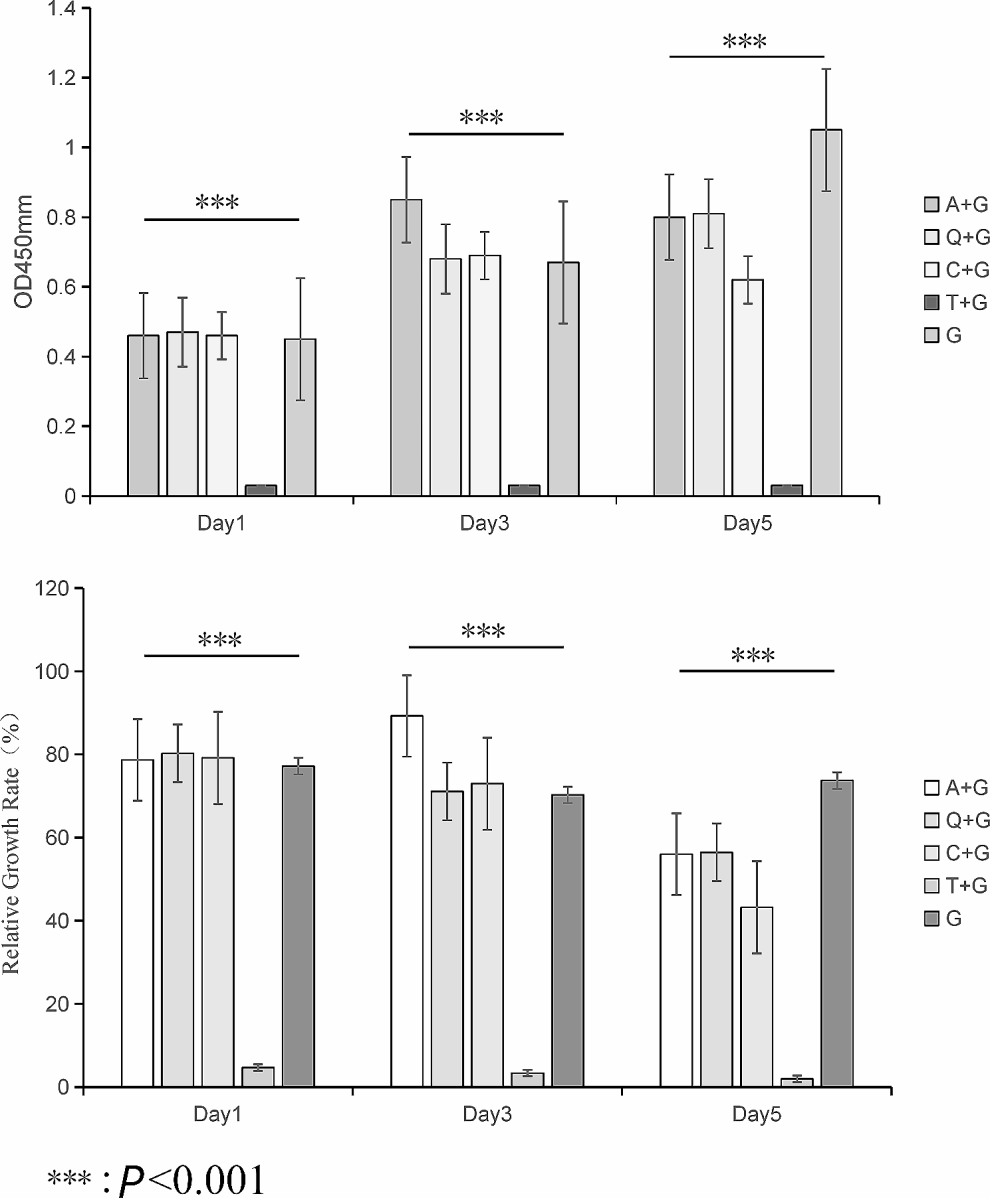
Comparison of the OD value at varying time intervals among groups treated with different drugs. Comparison of the RGR of cells within each group at varying time intervals. *Note* Denotations of each letter in Fig. 3: A: aloe-emodin, Q: quercetin, C: curcumin, T: tannic acid, G: gentamicin

We used repeated measures to compare the OD values of various drugs on different dates without considering concentration. As seen in the results, the main effect of drugs was significant (F = 17.39, *P* < 0.05). The OD value of tannic acid was significantly lower than that of other drugs. The main effect of duration was significant (F = 27.72, *P* < 0.05). The OD values in each drug group significantly decreased over time. Finally, the interaction effect was significant (F = 4.34, *P* < 0.05) (Fig. 3A and B).

We compared groups I, II, and V with the sole gentamicin control group with respect to cell OD value and RGR at different time points. We found that groups I, II, and V showed significant decreases in cell OD value and RGR, which were statistically significant (*P* < 0.05), and the most noticeable decrease occurred on the 5th day. In contrast, there was a gradual increase in cell OD values in the control group on days 1, 3, and 5, while the RGR was relatively consistent. Additionally, we observed higher cell OD values and RGR in groups V and VI when compared to group XXIV (a control group) at these time points.

When we compared the quercetin groups (groups VI, VII, and X) with the sole gentamicin control group (group XXIV), we found that groups VI, VII, and X showed a steady increase in the cell OD value and RGR. Among them, groups VII and X had significantly higher OD values and RGR on days 1 and 3 than group XXIV (*P* < 0.05) .

A comparison of the curcumin groups (groups XI, XII, and XV) with the sole gentamicin control group (group XXIV) revealed an increase in the cell OD value and RGR in groups XI, XII, and XV. Among them, groups XII had higher OD values and RGR at all the time points than group XXIV, while groups XI and XV had higher OD values and RGR on days 1 and 3 than group XXIV, with statistically significant differences (*P* < 0.05).

We compared the tannic acid groups (groups XVI, XVII, and XX) with the sole gentamicin control group (group XXIV). The results showed that groups XVI, XVII, and XX had a decrease in the cell OD value and RGR at all the time points, with all the OD values and RGR being significantly lower than those of group XXIV (*P* < 0.05).

## Discussion

Bacteria produce a biofilm during growth and reproduction, which exhibits resistance to antibiotics [14–17]. Several methods to disrupt bacterial biofilms have been discovered in recent years, with research on the role played by natural product extracts gaining popularity [18–20]. For instance, Dassanayake et al. suggested that allicin could enhance the bactericidal effect of vancomycin by binding to the SMARAMMs protein on the surface of *S. aureus* [21]. Shehzad et al. observed a significant increase in the antibacterial effect of gentamicin when combined with curcumin [22]. In this study, consistent with the experimental results of Lee et al., we found that curcumin increased the inhibition zone of gentamicin-loaded bone cement.

We also observed in our experiments that incorporating the four natural product extracts individually into the bone cement did not result in the formation of inhibition zones. This suggests that the natural product extracts, when used individually, may not have antibacterial effects. It is possible that the natural product extracts disrupt the biofilm formed on the bacterial surface during bacterial growth and reproduction, making it easier for antibiotics to come into contact with the bacterial surface. As a result, bacteria may exhibit increased sensitivity to antibiotics macroscopically [23]. 

Gentamicin is toxic to osteoblasts and can inhibit their bone-forming effectiveness. As reported by Sansonetti et al. [24]. , high doses of gentamicin can also trigger severe adverse reactions in the surrounding nerves. Therefore, one of the strategies to mitigate the suppression of osteoblast activity by ALBC is to enhance bacterial sensitivity to gentamicin, reduce the drug concentration required for sterilization, and consequently decrease the amount of antibiotics added to the antibacterial bone cement.

Aloe-emodin, quercetin, tannic acid, and curcumin have attracted attention in academic circles due to reports on their potential to inhibit the formation of bacterial biofilms [25]. Deng et al. observed that aloe-emodin exerts a significant inhibitory effect on the extracellular proteins and polysaccharides of the *S. aureus* biofilm [26]. Qi et al. reported that quercetin and tannic acid, when used in conjunction with antibiotics, exerted a notable antibacterial effect on methicillin-resistant *S. aureus* (MRSA). [27, 28].

In this study, we incorporated four natural product extracts into the bone cement in addition to gentamicin. We found that adding 5% natural product extracts reduced the quantity of gentamicin required to only 25–50% of the original dosage in order to achieve an equivalent antibacterial effect. This indicates that these natural product extracts, released from the bone cement, could enhance the antibacterial effect of gentamicin. The natural product extracts also effectively reduced the concentration of gentamicin in ALBC, thereby reducing its toxicity to surrounding tissue cells. Additionally, as indicated by the results of our CCK-8 experiments, except for the tannic acid group, the incorporation of natural product extracts did not notably impact the biocompatibility of the drug-loaded bone cement in the remaining test groups.

In summary, the four natural product extracts that we selected for this study—aloe-emodin, quercetin, tannic acid, and curcumin—were effective in enhancing the susceptibility of *S. aureus* to gentamicin in bone cement. This enhancement allowed for an equivalent bacteriostatic effect as before while concurrently reducing the dosage of gentamicin. A lower dosage of gentamicin implies better biocompatibility.

This study also has some limitations. (1) This was an in vitro study conducted in a laboratory environment, which may not necessarily reflect real-life clinical situations. We did not consider factors such as bodily fluids, physical activity, host responses, and antibiotic stability within the body. (2) The scope of this experiment was limited to testing only one common type of bacteria (*S. aureus*), and we did not include a wider range of infectious bacteria that are clinically common.

## Conclusion

In this study, we incorporated four natural product extract components—aloe-emodin, quercetin, tannic acid, and curcumin—into PMMA bone cement. We tested whether these extracts could augment the susceptibility of *S. aureus* to gentamicin in the bone cement. Our comparisons revealed that, in contrast to the bone cement solely employing gentamicin as the antibiotic, the bone cement with a combination of gentamicin and natural product extracts had notably enhanced antibacterial efficacy against *S. aureus*. Furthermore, the addition of 5% natural product extracts reduced the required dosage of gentamicin to only 25–50% of the original amount while maintaining an equivalent antibacterial effect. Therefore, aloe-emodin, quercetin, tannic acid, and curcumin could effectively reduce the gentamicin content in gentamicin-loaded bone cement without compromising its antibacterial effect. This reduction can potentially mitigate the adverse reactions triggered by gentamicin in the human body, demonstrating significant clinical potential for future applications.

## Data Availability

All data generated or analysed during this study are included in this article. Further enquiries can be directed to the corresponding author.
